# Neural representation of three-dimensional acoustic space in the human temporal lobe

**DOI:** 10.3389/fnhum.2015.00203

**Published:** 2015-04-16

**Authors:** Xiaolu Zhang, Qingtian Zhang, Xiaolin Hu, Bo Zhang

**Affiliations:** ^1^State Key Laboratory of Intelligent Technology and Systems, Tsinghua National Laboratory for Information Science and Technology (TNList), Department of Computer Science and Technology, Tsinghua UniversityBeijing, China; ^2^Center for Brain-Inspired Computing Research (CBICR), Tsinghua UniversityBeijing, China

**Keywords:** sound localization, functional MRI, multivariate pattern analysis, decoding, auditory spatial discrimination

## Abstract

Sound localization is an important function of the human brain, but the underlying cortical mechanisms remain unclear. In this study, we recorded auditory stimuli in three-dimensional space and then replayed the stimuli through earphones during functional magnetic resonance imaging (fMRI). By employing a machine learning algorithm, we successfully decoded sound location from the blood oxygenation level-dependent signals in the temporal lobe. Analysis of the data revealed that different cortical patterns were evoked by sounds from different locations. Specifically, discrimination of sound location along the abscissa axis evoked robust responses in the left posterior superior temporal gyrus (STG) and right mid-STG, discrimination along the elevation (EL) axis evoked robust responses in the left posterior middle temporal lobe (MTL) and right STG, and discrimination along the ordinate axis evoked robust responses in the left mid-MTL and right mid-STG. These results support a distributed representation of acoustic space in human cortex.

## Introduction

Sound localization plays an important role in everyday life. We can automatically identify the location of an acoustic target even in a noisy environment. This perception is mainly derived from interaural time differences (ITD), interaural level differences (ILD) and spectral cues (Blauert, 1997; Cohen and Knudsen, 1999; Grothe et al., 2010). These spatial cues are analyzed along the ascending auditory pathway (Thompson and Cortez, 1983; Wise and Irvine, 1985; Cohen and Knudsen, 1999; Grothe et al., 2010). Different areas in the temporal lobe have been shown to be sensitive to sound location (Recanzone, 2000; Tian et al., 2001; King et al., 2007; Lee and Middlebrooks, 2011), but the underlying mechanisms of auditory spatial processing in these areas remain unclear (Middlebrooks, 2002; Meyer et al., 2010; Salminen et al., 2010; Lewald and Getzmann, 2011; Schechtman et al., 2012).

It is reasonable to expect a topographic representation of sound location in the auditory cortex (Jeffress, 1948) because topographic representations, such as the tonotopic map found in primary auditory cortex (Merzenich et al., 1975) and the orientation map found in primary visual cortex (Hubel and Wiesel, 1959), seem to be a hallmark of cortical organization. However, such a representation has not been found even after intensive study (Cohen and Knudsen, 1999; Weeks et al., 1999; Lewald et al., 2000, 2008; Recanzone, 2000; Wessinger et al., 2001; Middlebrooks, 2002; Stecker and Middlebrooks, 2003; Zimmer et al., 2006; Deouell et al., 2007; Nakamoto et al., 2008; Altmann et al., 2009; Grothe et al., 2010; Lee and Middlebrooks, 2011). In fact, many physiological studies have found that auditory neurons in mammals have very broad tuning curves to sound location (spatial receptive fields typically span 150–180°) (Mickey and Middlebrooks, 2003; Stecker et al., 2005), although location can be decoded from a population of such neurons (Miller and Recanzone, 2009). Neuroimaging studies suggest that sound location is encoded in a distributed manner in the cortex (Weeks et al., 1999; Maeder et al., 2001; Zatorre et al., 2002; Brunetti et al., 2005; Barrett and Hall, 2006; Altmann et al., 2008; Smith et al., 2010; Kong et al., 2014).

Most of these studies examined the neural encoding mechanisms using sounds presented along the horizontal plane. However, in reality sounds may come from anywhere within the entirety of three-dimensional (3D) space. Furthermore, it is possible that the encoding mechanisms differ for sounds arising from different locations. For instance, many studies have shown that neurons in both hemispheres prefer contralateral stimulation (Woldorff et al., 1999; Mickey and Middlebrooks, 2003; Miller and Recanzone, 2009; Yao et al., 2013), but a similar encoding mechanism for sound locations above and below the horizontal plane cannot exist because we do not have an “up brain” or “down brain”. A small number of studies (Lewald et al., 2008; Lewald and Getzmann, 2011) have used stimulus locations beyond the horizontal plane, but the analyses were restricted to the ability of the brain to distinguish among sounds along the abscissa dimension. Another study (Pavani et al., 2002) presented stimuli along a horizontal line and two vertical lines located in front of subjects, but the aim was to study the encoding of sounds moving along these lines. An electrophysiological study investigated sound localization mechanisms in monkeys using speakers distributed in 3D space (Zhou and Wang, 2012) but focused on the level tolerance of spatial perception in single neurons. Furthermore, in electrophysiological experiments, one can only observe the activity of a small number of neurons at a time, which is insufficient for discovering patterns of cortical activation across large areas.

In this study, we explored the underlying mechanisms of spatial sound perception over all of 3D space using functional magnetic resonance imaging (fMRI) in human cortex. We first recorded sounds from speakers distributed evenly throughout acoustic space and then played the recorded sounds in the fMRI chamber during brain scanning. Multivariate pattern analysis (MVPA) revealed that sound locations could be discriminated using brain activity for three different conditions: left vs. right, up vs. down, and front vs. back. Moreover, the cortical activity that enabled decoding under the different conditions displayed different spatial patterns in the super temporal gyrus (STG) and middle temporal lobe (MTL).

## Materials and Methods

We planned to analyze the brain activity evoked by spatial sounds. First, we needed to decide what types of stimuli should be used. One strategy is to use synthetic stereo sounds (Maeder et al., 2001; Zimmer et al., 2006; Kong et al., 2014). However, such sounds lack subject-specific spectral localization cues due to differences in individual anatomy. Accordingly, we decided to use realistic spatial sounds originating from 3D space that were customized for each subject. This was achieved by playing sounds via loudspeakers positioned around each subject’s head and recording the stimuli via inner-aural microphones. These recorded stimuli were then delivered to the subject via stereo earphones during fMRI scanning. This subject-specific stimulus design eliminates the influence of different head and torso shapes on the perception of sound location, which should more faithfully preserve spatial cues for each subject. However, the recording techniques, properties of the earphones and other factors may degrade the quality of the stimuli. Three behavioral experiments were designed to test if the majority of the spatial cues had been preserved during fMRI scanning. See below for details.

### Subjects

Eight right-handed subjects (1 female, age from 21 to 26, mean age 23) with normal symmetric hearing abilities participated in the experiments. Normal symmetric hearing abilities were confirmed before the experiments by testing the pure tone thresholds (PTTs) for all the subjects (GSI AudioStar Pro, Guymark, UK). The recording of one additional subject was aborted due to subject discomfort. All subjects provided informed consent prior to participation. The experimental protocols were approved by the institutional review board of the Biomedical Imaging Research Center, Tsinghua University. All procedures adhered to the tenets of the Declaration of Helsinki.

### Behavioral Experiment Setup

**Figure 1:**
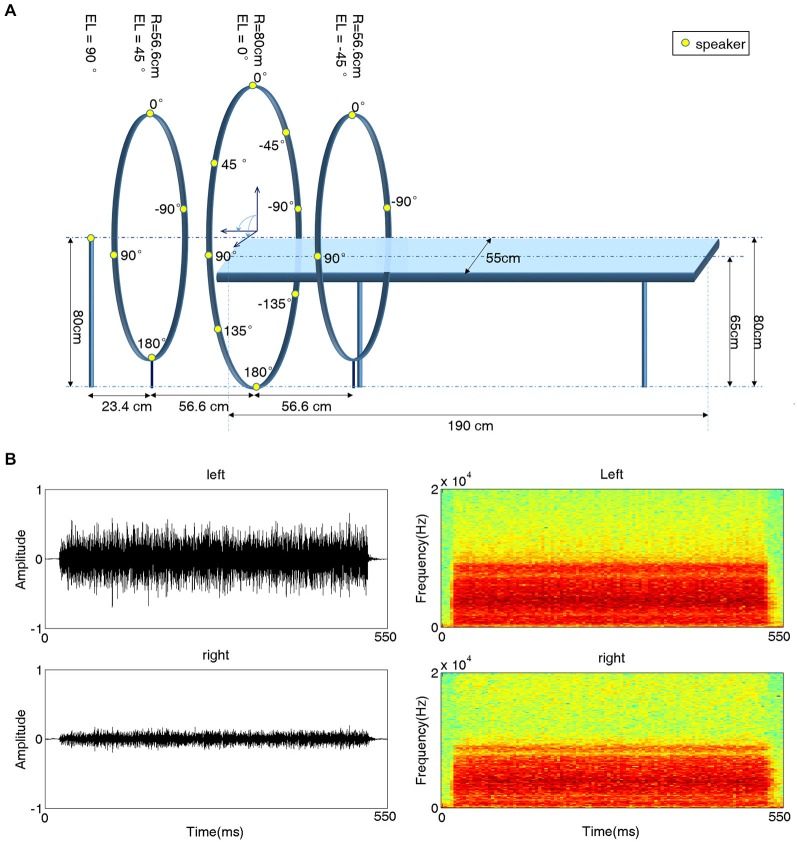
**Experimental setup for 3D acoustic stimulation. (A)** Schematic illustration of the experimental platform. A subject lay on a platform with sixteen speakers evenly distributed around his/her head. All speakers were placed on the surface of a sphere (radius 80 cm) centered at the subject’s head and immobilized facing the center. Fifteen speakers (yellow dots) were mounted on three iron hoops with radii of 56.6 cm, 80 cm and 56.6 cm and elevation (EL) angles of 45°, 0° and 45°, respectively. Four speakers were positioned at EL = 45°, eight speakers at EL = 0°, and three speakers at EL = −45°. azimuth (AZ) angles are indicated next to the speakers. The 16th speaker was positioned at EL 90° (on top of the subject’s head). **(B)** Waveforms (left) and spectrograms (right) of a sample stimulus recorded from one subject’s left (top) and right (bottom) ear for a sound presented at EL = 0° and AZ = −45°.

A behavioral experiment setup was built in a double-walled, sound-attenuating and echo-reduced chamber (IAC-1205A, Industrial Acoustics, UK). The setup contained a platform similar to the one used in subsequent fMRI scanning sessions. When subjects laid on this platform, sixteen speakers were evenly distributed around their heads along a spherical surface with a radius of 80 cm (Figure 1A). The position of a given speaker is specified in angles of azimuth (AZ) and elevation (EL). The speaker just in front of the subject’s eyes was located at AZ = 0° and EL = 0° (negative AZ values indicate positions to the left). One speaker was positioned just above the head of the subject (EL = 90°); four speakers were evenly positioned at EL = 45° with 90° horizontal spacing (AZ = 0°, ±90°, 180°); eight speakers were positioned at EL = 0° (AZ = 0°, ±45°, ±90°, ±135°, 180°); and three speakers were positioned at EL = −45° (AZ = 0°, ±90°). All speakers were immobilized facing the center of the sphere, i.e., the subject’s head.

### Acoustic Stimuli and Experimental Procedures

#### Behavioral Task

Each subject underwent three experiments in sequence, with either speaker-delivered sounds or earphone-delivered sounds. The procedures for these experiments are detailed below. In all experiments, the behavioral task was the same: immediately after the presentation of a stimulus, the subjects had to indicate their perception of the position of the stimulus by pressing one of three buttons (“left”, “middle”, or “right”) with their right hands. Here, “left” stimuli were defined as those originating from the speakers with negative AZ values (EL = 0°, ±45°), “right” stimuli were defined as those from the speakers with positive AZ values (EL = 0°, ±45°), and “middle” stimuli were defined as those from the speakers with zero AZ values (EL = 0°, ±45°) and the speaker with EL = 90°.

#### Experiment 1

Subjects lay on the platform in the acoustic chamber with their heads positioned at the center of the spherical surface where the 16 speakers were mounted. Mono stimuli were delivered to the speakers via a digital-to-analog interface (TDT, Tucker-Davis Technologies, Florida). A total of 80 (5 per speaker) stimuli were pseudo-randomly presented. The stimuli used in this experiment consisted of band-passed noise (20 Hz–12 kHz), with a duration of 500 ms and a sampling rate of 44.1 kHz.

#### Experiment 2

This experiment was the same as Experiment 1 except that the stimuli were the recorded sounds, which were subject-specific and were delivered through earphones. The sound recording procedure was as follows. Before the experiment, two inner-ear microphones were placed in the external auditory canal of each subject. Then, the stimuli used in Experiment 1 were played through the speakers. The microphones recorded the speaker-delivered sounds via a stereophonic preamplifier with a sampling rate of 44.1 kHz. Finally, the recorded stereo sounds were trimmed to 550-ms segments. The waveform and spectrogram of an example recorded stimulus are shown in Figure 1B.

#### Experiment 3

**Figure 2:**
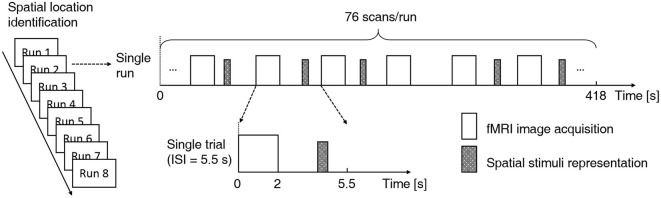
**Functional magnetic resonance imaging (fMRI) experimental design**. A fast event-related design and a sparse scanning protocol were adopted. Each subject participated in eight functional runs using the same experiment protocol. Each run consisted of 76 trials, and within each run, 64 stimulus trials (4 trials per sound location) and 12 null trials were randomly intermixed. Each trial began with 2 s of image acquisition, and the stimulus (if any) was presented in the subsequent 3.5 s (TR = 5.5 s). For stimulus trials, the onset of the 550-ms stimulus was jittered throughout the 2.5 s that followed image acquisition. A location identification task was conducted during functional scanning. Subjects were asked to indicate the location of each stimulus (left, middle or right) with a right-handed button press.

This was the fMRI scanning experiment, during which the same behavioral task was carried out. Subject-specific recorded stimuli (see Experiment 2 above) were delivered through MR-compatible electrodynamic earphones. The experiment started with a practice run in which several spatial stimulus sequences were delivered to familiarize the subjects with the fMRI environment. The practice run was followed by eight functional runs. Each run consisted of 76 trials. Of these trials, 64 were stimulus trials (4 trials per sound location) and 12 were “silent” trials (without stimulus presentation), which were randomly intermixed (Figure 2). Because the acoustic noise generated during image acquisition could interfere with the perception of sound location, the stimuli were delivered during scan intervals (Hall et al., 1999; Joanisse et al., 2007). Each trial began with 2 s of image acquisition, and the stimulus (if any) was presented in the subsequent 3.5 s (TR = 5.5 s). The 550-ms-long stimulus could begin at any point within the 2.5 s that followed image acquisition. Each run lasted 418 s. Short breaks were included between runs. The whole fMRI experiment lasted approximately 1 h. Subjects were asked to focus on the behavioral task and to ignore the machine noise. Note that subjects did not need to press buttons during the “silent” trials.

### MRI Acquisition

During Experiment 3 (described above), brain imaging was performed in a 3-Tesla MRI system (Philips; Achieva) with an 8-channel head coil at the Center for Biomedical Imaging Research of Tsinghua University. Each subject participated in two sets of scans. First, high-resolution anatomical image scans were obtained using a T1-weighted MPRAGE pulse sequence to generate anatomical images coregistered with the functional data. Anatomical images were acquired using 180 1-mm slices (256 * 256 matrix; 0.9 * 0.9 mm in-plane resolution; time repetition (TR) = 7.7 ms; time to echo (TE) = 3.8 ms; flip angle = 8°; field of view (FOV), 230 mm). Functional data were then acquired while subjects were performing the behavioral task. Functional images were obtained using a standard echo-planar imaging (EPI) sequence and 34 4-mm no gap slices (144 * 144 matrix; 1.56 * 1.56 mm in-plane resolution; TR = 5.5 s; TE = 28.7 ms; flip angle = 90°; field of view (FOV), 224 mm).

### fMRI Data Preprocessing

Functional brain volumes were analyzed using Statistical Parametric Mapping software (SPM8).^1^ Individual functional volumes were motion corrected through realignment to the first EPI image, coregistered with each subject’s anatomical image, spatially normalized into MNI space and resampled in 1.56 * 1.56 * 3 mm^3^ voxels. These preprocessed data were then analyzed using two complementary methods: voxel-wise general linear model (GLM) analysis and ROI-based MVPA.

### Univariate GLM Analysis

**Figure 3:**
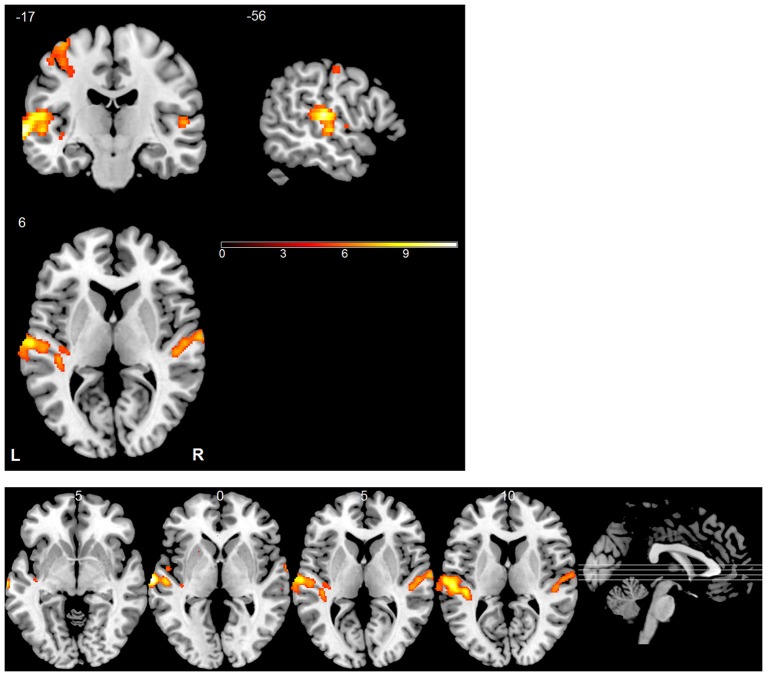
**Statistical parametric map of active regions using “stimulus vs. silent” contrast**. The significance level for activation was set at *p* < 0.05 (FDR corrected). The bottom panel shows a multi-slice orthogonal view.

A GLM whose repressors matched the time course of the experimental conditions was applied to each subject’s data to identify voxels activated by the stimuli. The predicted activation time course was modeled as a “gamma” function convolved with the canonical hemodynamic response function. Voxel-vise parameter estimation was carried out according to the GLM. To improve the signal-to-noise ratio, a Gaussian kernel (5 * 5 * 5 mm^3^) was applied to the normalized data prior to the GLM analysis. For each subject, an activation map was generated by contrasting stimulus trials with silent trials (Figure 3). Similarly, we analyzed differences between the blood oxygenation level dependent (BOLD) patterns using GLM by contrasting different sound locations (left vs. right, up vs. down and front vs. back) but did not find any significant effects. In all of these analyses, the statistical threshold was set to *p* < 0.05, corrected by the false discovery rate (FDR). MRIcron was used to display activation maps on a standard brain template (Rorden and Brett, 2000).

### MVPA

Preprocessed functional data for each run were separately fit with a GLM. At every voxel, a GLM was applied with one predictor coding for the stimulus response and one linear predictor accounting for a within-trial linear trend (Friston et al., 2007; Kay et al., 2008). The regression coefficient (beta) of the stimulus response was taken to represent this stimulus at this voxel (for convenience this coefficient is also called the “response” of the stimulus at the voxel). However, we did not fit the 64 stimuli individually. Instead, the four stimuli from the same location in each run were combined together, which resulted in 16 responses. Therefore, a total number of 128 brain response images were obtained for each subject’s eight runs. Note that the data were not smoothed for regression.

A linear support vector machine (SVM; Burges, 1998) was trained to classify the responses into different categories based on the locations of the stimuli: left vs. right (abscissa discrimination), up vs. down (EL discrimination) and front vs. back (ordinate discrimination). A leave-one-run-out cross-validation was performed for each subject. In other words, the brain images obtained from all but one of the functional runs were used to train the classifier, and the run excluded from training was used for testing. This process was repeated for each run in turn. The average accuracy over all testing runs was computed.

Due to the high resolution of fMRI, each brain image contained a massive number of voxels. A two-step approach was used to reduce the number of voxels to facilitate the multivariate classification as described above. First, a mask was obtained by combining Brodmann areas 41, 42 and 22 in each hemisphere, and a region of interest (ROI) was defined by smoothing this mask with a Gaussian kernel (5 * 5 * 5 mm^3^). Thus, all activated voxels in the univariate analysis except those in the left motor cortex were included in this ROI, which mainly consist of the superior temporal lobe and the superior part the MTL. Then, for each discrimination condition, a permutation test-based approach was employed to further reduce the number of voxels (Nichols and Holmes, 2002), which is described as follows. For any discrimination condition, the class labels of the training set were randomly permutated 2000 times. Accordingly, 2000 linear SVMs were trained with these labels. For each voxel, a probability distribution of its linear weight in the classifier was estimated. We found that a Gaussian distribution was sufficient to fit the data. Based on these probability distributions, we tested the null hypothesis of no relationship between the voxel’s response and its true class label. If the weight of a voxel determined using the true location labels lies far outside the major mass of the distribution, as indicated by a small *p*-value, then the null hypothesis is unlikely to be valid, and the voxel can be treated as “active”, i.e., relevant for this discrimination condition. This is a multivariate analysis approach. To prevent over-fitting only training data were used in the permutation test.

Note that the voxel selection procedure for each discrimination condition was performed separately for each of the eight runs in each subject. A voxel selected in more than four runs in a single subject was defined as an effective voxel (EV) for that discrimination condition. Selecting an EV in a single subject can be considered as a Bernoulli trial with probability *p* for successful trials. In this study p was assigned less than 0.05 by the permutation test. If we treat the exact number of subjects in which the same voxel is successfully selected as a random variable *X*, then *X* follows a binomial distribution and *P*(*X* = *k*) = *C*(8, *k*) * *p^k^* * (1 – *p*)^(8–*k*)^. The probability of selecting the same voxel in three or more subjects is *P*(*X* ≥ 3) = 1 – *P*(*X* = 0) – *P*(*X* = 1) − *P*(*X* = 2). It is easy to verify that this function monotonically decreases if *p* decreases. Since *p* < 0.05, we have *P*(*X* ≥ 3) < 0.58 * 10^−3^. In this sense, we defined an EV selected in three out of eight subjects as a Significant Effective Voxel (SEV).

## Results

### Behavioral Results

In Experiments 1, 2 and 3, all subjects showed consistent performance and were able to precisely judge sound location. The mean accuracy of each experiment was 99.53% (SD = 0.013), 98.28% (SD = 0.021) and 98.14% (SD = 0.018), respectively. These high accuracies indicated that: (1) all subjects had normal hearing abilities and could accurately discriminate the location of spatial sounds; and (2) the recorded 3D stimuli did not degrade important auditory cues for location perception during fMRI scanning (Møller, 1992; Grothe et al., 2010).

### Univariate fMRI Data Analysis

During Experiment 3, functional images were collected for each subject while they were listening to the individually recorded stimuli. Consistent with previous studies (Wessinger et al., 2001; Pavani et al., 2002; Zimmer et al., 2006; Deouell et al., 2007; Lewald et al., 2008), univariate analysis revealed that the stimuli, when combined, evoked significant fMRI responses across the auditory cortex bilaterally (Figure 3). The largest and most robust activation was observed in the STG. The left motor cortex was also conspicuously activated due to the right-hand responses.

We were interested in whether the sounds from different locations could elicit different BOLD response patterns in the cortex; therefore, we constructed three contrast conditions according to the sound’s location relative to the subject: left vs. right, up vs. down and front vs. back. However, univariate analyses did not yield significant difference in any of these contrast conditions (threshold 0.05, FDR corrected).

### Decoding Sound Location via MVPA

Compared with univariate analysis, MVPA methods enable the study of the spatial pattern of brain activity across many voxels simultaneously and boost the detection sensitivity of cognitive states (De Martino et al., 2008; Formisano et al., 2008a,b; Mitchell et al., 2008; Pereira et al., 2009; Meyer et al., 2010). We thus performed MVPA to decode sound location from the brain activity. Specifically, we trained a linear SVM classifier on the BOLD signals to discriminate the location of sounds in each of the three conditions: left vs. right, up vs. down, and front vs. back. Before applying the classifier, a two-step approach was used to select relevant features (see Section Materials and Methods for details). A leave-one-run-out cross-validation was performed during classification for each subject, and the prediction accuracy was defined as the average testing accuracy over eight validations.

**Figure 4:**
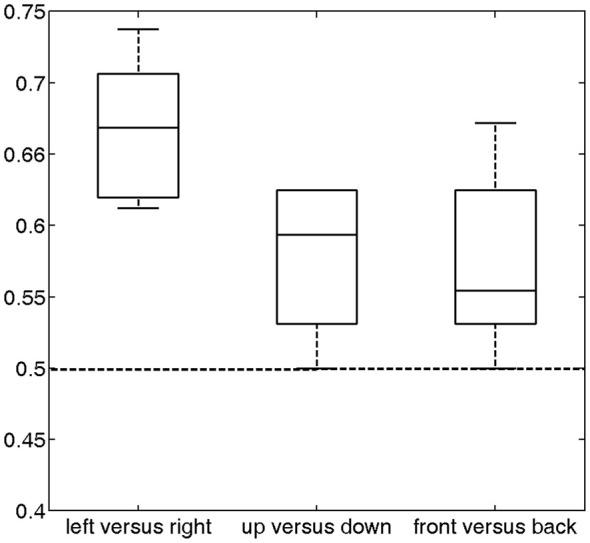
**Classification accuracy across all subjects (median and distribution) for the three discrimination conditions (left vs. right, up vs. down and front vs. back)**. For these conditions, the average accuracies were 66.74% (*p* = 2.4 * 10^−5^), 57.81% (*p* = 0.0033) and 57.42% (*p* = 0.012), respectively, which were significantly higher than chance (50%, dashed horizontal line).

The prediction accuracy over all subjects for left vs. right was 66.74% (Figure 4), which was significantly higher than the chance level (50%) (*p* = 2.4 * 10^−5^). The prediction accuracies for up vs. down and front vs. back were 57.81% and 57.42%, respectively. Each of these accuracies was significantly higher than the chance level (*p* < 0.012). These results indicate that the temporal lobe exhibits distinguishable response patterns in response to different sound locations. The much lower accuracies for front vs. back and up vs. down than left vs. right suggests that the cortex may encode sound location in different spatial dimensions using different mechanisms.

### Spatial Patterns of BOLD Signals in the Auditory Cortex

We investigated the layout and consistency of spatial patterns in the cortex across subjects, which enabled the classification of sound locations. Voxels effective for discriminating the specific classification conditions in a single subject was defined as EVs. When an EV appeared in three or more subjects, it was defined as a SEV. The SEVs were strongly correlated with the corresponding discrimination condition (see Section Materials and Methods).

**Figure 5:**
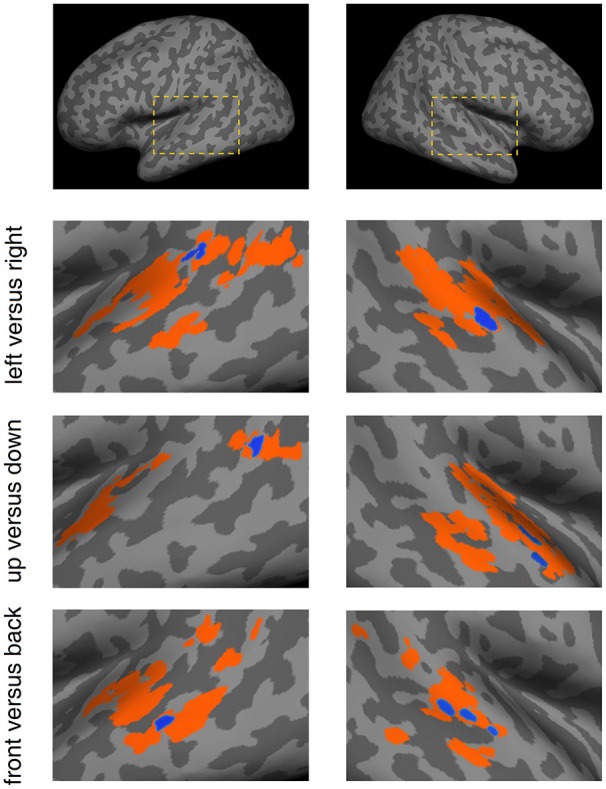
**Spatial patterns of effective voxels (EV) within the temporal lobe for the different discrimination conditions**. For each condition, the voxels displayed in color were obtained from the permutation test-based voxel selection procedure. Orange signifies voxels selected in at least one subject and blue signifies voxels selected in three or more subjects. Blue voxels were referred to as Significant Effective Voxels (SEVs). The dashed boxes in the top panels depict the location of these results in the brain.

The SEVs for each discrimination condition were projected onto a standard inflated cortical surface (Figure 5). They exhibited distinct patterns that corresponded to the different discrimination conditions. For the left vs. right condition, SEVs were concentrated in the left posterior STG and right mid-STG. For the up vs. down condition, SEVs were concentrated in the left posterior-MTL and right STG. For the front vs. back condition, SEVs were concentrated in the left mid-MTL and right mid-STG.

## Discussion

In this study, we used machine learning techniques to study brain activation patterns as a function of the location of auditory objects. We found that the 3D locations of stimuli could be decoded from cortical activity. Voxels exhibiting robust activity formed distinct patterns in the temporal lobe that were effective in discriminating different sound locations across subjects. Abscissa discrimination (left vs. right) was more related to the left posterior STG and right mid-STG. Ordinate discrimination (front vs. back) was more related to the left posterior-MTL and right STG. Finally, EL discrimination (up vs. down) was more related to the left mid-MTL and right mid-STG.

Many fMRI studies (Wessinger et al., 2001; Pavani et al., 2002; Zimmer et al., 2006; Deouell et al., 2007; Lewald et al., 2008) have shown that cortical activity is related to sound location along the abscissa dimension. Our results further indicate that cortical activity contains sufficient information for the classification of sounds along the abscissas dimension. Similar results have been obtained with single cell recordings and a population decoding method (Miller and Recanzone, 2009). What is new in our results is that we have used cortical activity to classify sounds along the ordinate and EL dimensions. However, classification accuracy for ordinate and EL discrimination was significantly lower than for abscissa discrimination. One possibility is that the behavioral task (indicating if the stimulus was from the left, middle or right of head) performed during fMRI scanning caused the difference in accuracy because the subjects attended to variation in the sound that were present along the abscissa dimension. However, that should not be the major factor underlying the difference. In fact, our decoding results are consistent with previous behavioral studies (Makous and Middlebrooks, 1990; Carlile et al., 1997) that reported lower localization errors for AZ discrimination than for EL discrimination in a natural auditory environment. Precise discrimination for up vs. down and front vs. back usually depends on visual assistance and head motion (Thurlow and Runge, 1967; Lewald et al., 2000), which were absent in our experiments.

The across-subject SEVs for discriminating sound location along the different dimensions exhibited different spatial patterns. The voxels effective for abscissa discrimination were mainly located in the STG bilaterally, which is consistent with previous neuroimaging findings (Brunetti et al., 2005; Zimmer and Macaluso, 2005; Zimmer et al., 2006; Lewald et al., 2008; Ahveninen et al., 2013). The across-subject SEVs for EL discrimination and ordinate discrimination were primarily located in the left MTL and right STG. The possible reason for the involvement of the MTL in the EL condition and ordinate condition but not in the horizontal condition is that precise localization of sounds in those two dimensions usually requires visual assistance due to the up-down confusions and cone of confusions (Carlile et al., 1997; Algazi et al., 2001; Grothe et al., 2010), while the temporal lobe is suggested to be involved in auditory-visual spatial integration (Kaas and Hackett, 2000; Zimmer et al., 2006; Lewald et al., 2008). The implications of different spatial patterns evoked by different conditions revealed in this study are unclear and need further investigation. Inconsistent with our results, a previous fMRI study (Pavani et al., 2002) did not reveal differences in brain activity for sounds moving in the vertical plane and horizontal plane. This discrepancy might be due to the different types of stimuli (stationary vs. moving) or the analysis methods (multivariate vs. univariate) used.

In conclusion, our results support the hypothesis that auditory spatial information is represented in the cortex in a distributed, not topographic, manner. The presence of distinct spatial patterns of significant EVs under different discrimination conditions suggests dissimilar processing mechanisms for sound location along different dimensions of 3D acoustic space.

## Conflict of Interest Statement

The authors declare that the research was conducted in the absence of any commercial or financial relationships that could be construed as a potential conflict of interest.

## Footnotes

^1^http://www.fil.ion.ucl.ac.uk/spm/software/spm8

## References

[B1] Ahveninen J. Huang S. Nummenmaa A. Belliveau J. W. Hung A. Y. Jääskeläinen I. P.. (2013). Evidence for distinct human auditory cortex regions for sound location versus identity processing. Nat. Commun. 4:2585. 10.1038/ncomms3585 24121634 PMC3932554

[B2] Algazi V. R. Avendano C. Duda R. O. (2001). Elevation localization and head-related transfer function analysis at low frequencies. J. Acoust. Soc. Am. 109, 1110–1122. 10.1121/1.1349185 11303925

[B3] Altmann C. F. Henning M. Döring M. K. Kaiser J. (2008). Effects of feature-selective attention on auditory pattern and location processing. Neuroimage 41, 69–79. 10.1016/j.neuroimage.2008.02.013 18378168

[B4] Altmann C. F. Wilczek E. Kaiser J. (2009). Processing of auditory location changes after horizontal head rotation. J. Neurosci. 29, 13074–13078. 10.1523/JNEUROSCI.1708-09.2009 19828820 PMC6665287

[B5] Barrett D. J. K. Hall D. A. (2006). Response preferences for “what” and “where” in human non-primary auditory cortex. Neuroimage 32, 968–977. 10.1016/j.neuroimage.2006.03.050 16733092

[B6] Blauert J. (1997). Spatial Hearing: The Psychophysics of Human Sound Localization. London: The MIT Press.

[B7] Brunetti M. Belardinelli P. Caulo M. Del Gratta C. Della Penna S. Ferretti A.. (2005). Human brain activation during passive listening to sounds from different locations: an fMRI and MEG study. Hum. Brain Mapp. 26, 251–261. 10.1002/hbm.20164 15954141 PMC6871706

[B8] Burges C. C. J. C. (1998). A tutorial on support vector machines for pattern recognition. Data Min. Knowl. Discov. 2, 121–167 10.1023/A:1009715923555

[B9] Carlile S. Leong P. Hyams S. (1997). The nature and distribution of errors in sound localization by human listeners. Hear. Res. 114, 179–196. 10.1016/s0378-5955(97)00161-5 9447931

[B10] Cohen Y. E. Knudsen E. I. (1999). Maps versus clusters: different representations of auditory space in the midbrain and forebrain. Trends Neurosci. 22, 128–135. 10.1016/s0166-2236(98)01295-8 10199638

[B11] De Martino F. Valente G. Staeren N. Ashburner J. Goebel R. Formisano E. (2008). Combining multivariate voxel selection and support vector machines for mapping and classification of fMRI spatial patterns. Neuroimage 43, 44–58. 10.1016/j.neuroimage.2008.06.037 18672070

[B12] Deouell L. Y. Heller A. S. Malach R. D’Esposito M. Knight R. T. (2007). Cerebral responses to change in spatial location of unattended sounds. Neuron 55, 985–996. 10.1016/j.neuron.2007.08.019 17880900

[B13] Formisano E. De Martino F. Bonte M. Goebel R. (2008a). “Who” is saying “what”? Brain-based decoding of human voice and speech. Science 322, 970–973. 10.1126/science.1164318 18988858

[B14] Formisano E. De Martino F. Valente G. (2008b). Multivariate analysis of fMRI time series: classification and regression of brain responses using machine learning. Magn. Reson. Imaging 26, 921–934. 10.1016/j.mri.2008.01.052 18508219

[B15] Friston K. J. Ashburner J. T. Kiebel S. J. Nichols T. E. Penny W. D. (2007). Statistical Parametric Mapping: The Analysis of Functional Brain Images. London: Academic Press.

[B16] Grothe B. Pecka M. McAlpine D. (2010). Mechanisms of sound localization in mammals. Physiol. Rev. 90, 983–1012. 10.1152/physrev.00026.2009 20664077

[B59] Hall D. A. Haggard M. P. Akeroyd M. A. Palmer A. R. Summerfield A. Q. Elliott M. R.. (1999). “Sparse” temporal sampling in auditory fMRI. Hum. Brain Mapp. 7, 213–223. 10.1002/(SICI)1097-0193(1999)7:3<213::AID-HBM5>3.0.CO;2-N 10194620 PMC6873323

[B17] Hubel D. H. Wiesel T. N. (1959). Receptive fields of single neurones in the cat’s striate cortex. J. Physiol. 148, 574–591. 10.1113/jphysiol.1959.sp006308 14403679 PMC1363130

[B18] Jeffress L. A. (1948). A place theory of sound localization. J. Comp. Physiol. Psychol. 41, 35–39. 10.1037/h0061495 18904764

[B19] Joanisse M. F. Zevin J. D. McCandliss B. D. (2007). Brain mechanisms implicated in the preattentive categorization of speech sounds revealed using FMRI and a short-interval habituation trial paradigm. Cereb. Cortex 17, 2084–2093. 10.1093/cercor/bhl124 17138597

[B20] Kaas J. H. Hackett T. A. (2000). Subdivisions of auditory cortex and processing streams in primates. Proc. Natl. Acad. Sci. U S A 97, 11793–11799. 10.1073/pnas.97.22.11793 11050211 PMC34351

[B21] Kay K. N. Naselaris T. Prenger R. J. Gallant J. L. (2008). Identifying natural images from human brain activity. Nature 452, 352–355. 10.1038/nature06713 18322462 PMC3556484

[B22] King A. J. Bajo V. M. Bizley J. K. Campbell R. A. A. Nodal F. R. Schulz A. L.. (2007). Physiological and behavioral studies of spatial coding in the auditory cortex. Hear. Res. 229, 106–115. 10.1016/j.heares.2007.01.001 17314017 PMC7116512

[B23] Kong L. Michalka S. W. Rosen M. L. Sheremata S. L. Swisher J. D. Shinn-Cunningham B. G.. (2014). Auditory spatial attention representations in the human cerebral cortex. Cereb. Cortex 24, 773–784. 10.1093/cercor/bhs359 23180753 PMC3920769

[B24] Lee C. C. Middlebrooks J. C. (2011). Auditory cortex spatial sensitivity sharpens during task performance. Nat. Neurosci. 14, 108–114. 10.1038/nn.2713 21151120 PMC3076022

[B25] Lewald J. Dörrscheidt G. J. Ehrenstein W. H. (2000). Sound localization with eccentric head position. Behav. Brain Res. 108, 105–125. 10.1016/s0166-4328(99)00141-2 10701655

[B26] Lewald J. Getzmann S. (2011). When and where of auditory spatial processing in cortex: a novel approach using electrotomography. PLoS One 6:e25146. 10.1371/journal.pone.0025146 21949873 PMC3176323

[B27] Lewald J. Riederer K. A. J. Lentz T. Meister I. G. (2008). Processing of sound location in human cortex. Eur. J. Neurosci. 27, 1261–1270. 10.1111/j.1460-9568.2008.06094.x 18364040

[B28] Maeder P. P. Meuli R. A. Adriani M. Bellmann A. Fornari E. Thiran J. P.. (2001). Distinct pathways involved in sound recognition and localization: a human fMRI study. Neuroimage 14, 802–816. 10.1006/nimg.2001.0888 11554799

[B29] Makous J. C. Middlebrooks J. C. (1990). Two-dimensional sound localization by human listeners. J. Acoust. Soc. Am. 87, 2188–2200. 10.1121/1.399186 2348023

[B30] Merzenich M. M. Knight P. L. Roth G. L. (1975). Representation of cochlea within primary auditory cortex in the cat. J. Neurophysiol. 38, 231–249. 1092814 10.1152/jn.1975.38.2.231

[B31] Meyer K. Kaplan J. T. Essex R. Webber C. Damasio H. Damasio A. (2010). Predicting visual stimuli on the basis of activity in auditory cortices. Nat. Neurosci. 13, 667–668. 10.1038/nn.2533 20436482

[B32] Mickey B. J. Middlebrooks J. C. (2003). Representation of auditory space by cortical neurons in awake cats. J. Neurosci. 23, 8649–8663. 14507964 10.1523/JNEUROSCI.23-25-08649.2003 PMC6740412

[B33] Middlebrooks J. C. (2002). Auditory space processing: here, there or everywhere? Nat. Neurosci. 5, 824–826. 10.1038/nn0902-824 12196806

[B34] Miller L. M. Recanzone G. H. (2009). Populations of auditory cortical neurons can accurately encode acoustic space across stimulus intensity. Proc. Natl. Acad. Sci. U S A 106, 5931–5935. 10.1073/pnas.0901023106 19321750 PMC2667094

[B35] Mitchell T. M. Shinkareva S. V. Carlson A. Chang K. M. Malave V. L. Mason R. A.. (2008). Predicting human brain activity associated with the meanings of nouns. Science 320, 1191–1195. 10.1126/science.1152876 18511683

[B36] Møller H. (1992). Fundamentals of binaural technology. Appl. Acoust. 36, 171–218 10.1016/0003-682x(92)90046-u

[B37] Nakamoto K. T. Jones S. J. Palmer A. R. (2008). Descending projections from auditory cortex modulate sensitivity in the midbrain to cues for spatial position. J. Neurophysiol. 99, 2347–2356. 10.1152/jn.01326.2007 18385487

[B38] Nichols T. E. Holmes A. P. (2002). Nonparametric permutation tests for functional neuroimaging: a primer with examples. Hum. Brain Mapp. 15, 1–25. 10.1002/hbm.1058 11747097 PMC6871862

[B39] Pavani F. Macaluso E. Warren J. D. Driver J. Griffiths T. D. (2002). A common cortical substrate activated by horizontal and vertical sound movement in the human brain. Curr. Biol. 12, 1584–1590. 10.1016/s0960-9822(02)01143-0 12372250

[B40] Pereira F. Mitchell T. Botvinick M. (2009). Machine learning classifiers and fMRI: a tutorial overview. Neuroimage 45, S199–S209. 10.1016/j.neuroimage.2008.11.007 19070668 PMC2892746

[B41] Recanzone G. H. (2000). Spatial processing in the auditory cortex of the macaque monkey. Proc. Natl. Acad. Sci. U S A 97, 11829–11835. 10.1073/pnas.97.22.11829 11050216 PMC34356

[B42] Rorden C. Brett M. (2000). Stereotaxic display of brain lesions. Behav. Neurol. 12, 191–200. 10.1155/2000/421719 11568431

[B43] Salminen N. H. Tiitinen H. Miettinen I. Alku P. May P. J. C. (2010). Asymmetrical representation of auditory space in human cortex. Brain Res. 1306, 93–99. 10.1016/j.brainres.2009.09.095 19799877

[B44] Schechtman E. Shrem T. Deouell L. Y. (2012). Spatial localization of auditory stimuli in human auditory cortex is based on both head-independent and head-centered coordinate systems. J. Neurosci. 32, 13501–13509. 10.1523/jneurosci.1315-12.2012 23015439 PMC6621373

[B45] Smith D. V. Davis B. Niu K. Healy E. W. Bonilha L. Fridriksson J.. (2010). Spatial attention evokes similar activation patterns for visual and auditory stimuli. J. Cogn. Neurosci. 22, 347–361. 10.1162/jocn.2009.21241 19400684 PMC2846529

[B46] Stecker G. C. Harrington I. A. Middlebrooks J. C. (2005). Location coding by opponent neural populations in the auditory cortex. PLoS Biol. 3:e78. 10.1371/journal.pbio.0030078 15736980 PMC1044834

[B47] Stecker G. C. Middlebrooks J. C. (2003). Distributed coding of sound locations in the auditory cortex. Biol. Cybern. 89, 341–349. 10.1007/s00422-003-0439-1 14669014

[B48] Thompson G. C. Cortez A. M. (1983). The inability of squirrel monkeys to localize sound after unilateral ablation of auditory cortex. Behav. Brain Res. 8, 211–216. 10.1016/0166-4328(83)90055-4 6860463

[B49] Thurlow W. R. Runge P. S. (1967). Effect of induced head movements on localization of direction of sounds. J. Acoust. Soc. Am. 42, 480–488. 10.1121/1.1910604 6075941

[B50] Tian B. Reser D. Durham A. Kustov A. Rauschecker J. P. (2001). Functional specialization in rhesus monkey auditory cortex. Science 292, 290–293. 10.1126/science.1058911 11303104

[B51] Weeks R. A. Aziz-Sultan A. Bushara K. O. Tian B. Wessinger C. M. Dang N.. (1999). A PET study of human auditory spatial processing. Neurosci. Lett. 262, 155–158. 10.1016/s0304-3940(99)00062-2 10218879

[B52] Wessinger C. M. VanMeter J. Tian B. Van Lare J. Pekar J. Rauschecker J. P. (2001). Hierarchical organization of the human auditory cortex revealed by functional magnetic resonance imaging. J. Cogn. Neurosci. 13, 1–7. 10.1162/089892901564108 11224904

[B53] Wise L. Z. Irvine D. R. (1985). Topographic organization of interaural intensity difference sensitivity in deep layers of cat superior colliculus: implications for auditory spatial representation. J. Neurophysiol. 54, 185–211. 4031984 10.1152/jn.1985.54.2.185

[B54] Woldorff M. G. Tempelmann C. Fell J. Tegeler C. Gaschler-Markefski B. Hinrichs H.. (1999). Lateralized auditory spatial perception and the contralaterality of cortical processing as studied with functional magnetic resonance imaging and magnetoencephalography. Hum. Brain Mapp. 7, 49–66. 10.1002/(sici)1097-0193(1999)7:1<49::aid-hbm5>3.3.co;2-a 9882090 PMC6873307

[B55] Yao J. D. Bremen P. Middlebrooks J. C. (2013). Rat primary auditory cortex is tuned exclusively to the contralateral hemifield. J. Neurophysiol. 110, 2140–2151. 10.1152/jn.00219.2013 23945782 PMC3841933

[B60] Zatorre R. J. Bouffard M. Ahad P. Belin P. (2002). Where is “where” in the human auditory cortex? Nat. Neurosci. 5, 905–909. 10.1038/nn904 12195426

[B56] Zhou Y. Wang X. (2012). Level dependence of spatial processing in the primate auditory cortex. J. Neurophysiol. 108, 810–826. 10.1152/jn.00500.2011 22592309 PMC3424089

[B57] Zimmer U. Lewald J. Erb M. Karnath H. O. (2006). Processing of auditory spatial cues in human cortex: an fMRI study. Neuropsychologia 44, 454–461. 10.1016/j.neuropsychologia.2005.05.021 16038950

[B58] Zimmer U. Macaluso E. (2005). High binaural coherence determines successful sound localization and increased activitiy in posterior auditory areas. Neuron 47, 893–905. 10.1016/j.neuron.2005.07.019 16157283

